# Exploring Substance Use Tweets of Youth in the United States: Mixed Methods Study

**DOI:** 10.2196/16191

**Published:** 2020-03-26

**Authors:** Robin C Stevens, Bridgette M Brawner, Elissa Kranzler, Salvatore Giorgi, Elizabeth Lazarus, Maramawit Abera, Sarah Huang, Lyle Ungar

**Affiliations:** 1 Department of Family and Community Health University of Pennsylvania School of Nursing Philadelphia, PA United States; 2 University of Pennsylvania School of Nursing Philadelphia, PA United States; 3 Wharton Risk Management and Decision Processes Center Philadelphia, PA United States; 4 Department of Computer and Information Science Philadelphia, PA United States; 5 Crescenz Veterans Affairs Medical Center Philadelphia, PA United States

**Keywords:** social media, illicit drug, youth, adolescent

## Abstract

**Background:**

Substance use by youth remains a significant public health concern. Social media provides the opportunity to discuss and display substance use–related beliefs and behaviors, suggesting that the act of posting drug-related content, or viewing posted content, may influence substance use in youth. This aligns with empirically supported theories, which posit that behavior is influenced by perceptions of normative behavior. Nevertheless, few studies have explored the content of posts by youth related to substance use.

**Objective:**

This study aimed to identify the beliefs and behaviors of youth related to substance use by characterizing the content of youths’ drug-related tweets. Using a sequential explanatory mixed methods approach, we sampled drug-relevant tweets and qualitatively examined their content.

**Methods:**

We used natural language processing to determine the frequency of drug-related words in public tweets (from 2011 to 2015) among youth Twitter users geolocated to Pennsylvania. We limited our sample by age (13-24 years), yielding approximately 23 million tweets from 20,112 users. We developed a list of drug-related keywords and phrases and selected a random sample of tweets with the most commonly used keywords to identify themes (n=249).

**Results:**

We identified two broad classes of emergent themes: functional themes and relational themes. *Functional* themes included posts that explicated a function of drugs in one’s life, with subthemes indicative of *pride*, *longing*, *coping*, and *reminiscing* as they relate to drug use and effects. *Relational* themes emphasized a relational nature of substance use, capturing substance use as a part of social relationships, with subthemes indicative of drug-related *identity* and *companionship*. We also identified topical areas in tweets related to drug use, including reference to *polysubstance use*, *pop culture*, and *antidrug* content. Across the tweets, the themes of *pride* (63/249, 25.3%) and *longing* (39/249, 15.7%) were the most popular. Most tweets that expressed *pride* (46/63, 73%) were explicitly related to marijuana. Nearly half of the tweets on *coping* (17/36, 47%) were related to prescription drugs. Very few of the tweets contained *antidrug* content (9/249, 3.6%).

**Conclusions:**

Data integration indicates that drugs are typically discussed in a positive manner, with content largely reflective of functional and relational patterns of use. The dissemination of this information, coupled with the relative absence of antidrug content, may influence youth such that they perceive drug use as normative and justified. Strategies to address the underlying causes of drug use (eg, coping with stressors) and engage antidrug messaging on social media may reduce normative perceptions and associated behaviors among youth. The findings of this study warrant research to further examine the effects of this content on beliefs and behaviors and to identify ways to leverage social media to decrease substance use in this population.

## Introduction

### Background

Despite previous decline in alcohol and drug use among youth, the rates of substance use have generally plateaued in recent years [1]. Estimates indicate that 62.5% of underage alcohol users are binge alcohol users, 1.6 million youth aged between 12 and 17 years used marijuana in the past month, and 7.3% of youth aged between 18 and 25 years misused opioids (eg, hydrocodone and oxycodone) in the past year [2]. This is a significant public health concern given that substance use, particularly early in life, is associated with a host of negative outcomes such as increased sexual risk behavior [3], negative academic outcomes, and increased risk of substance abuse later in life [4,5].

Social media use has increased dramatically over the past decade, with near-ubiquitous use among adolescents and young adults [6,7]. At least 85% of adolescents use one or more of the several popular social media platforms (eg, YouTube, Instagram, and Snapchat) [6], and 88% of young adults (aged 18-29 years) report using any form of social media [7]. Compared with other age groups, youth report the highest rates of use within and across social media platforms, noting that they use social media to connect with friends and family, to obtain news and information, for entertainment purposes, and as a space for self-expression [6,7]. With such high levels of Web engagement and diverse usage patterns, social media has drastically changed how information, both in general and specifically about risk-related behaviors (eg, alcohol and other substance use), is received by and exchanged among youth [8,9].

Youth use social media to discuss and display substance use behaviors [10-15], which have been linked to their behaviors offline [16]. Substantial research has demonstrated associations between substance-related social media engagement and substance use behaviors in real life [12,15,17,18], suggesting that the act of posting substance-related content, or viewing such content posted by others, may influence substance use in youth. This potential model of effects aligns with empirically supported theories of behavior change, which posit that risk behavior adoption is influenced by behavioral modeling and perceived norms (eg, perceptions of what one’s peers are doing) [19,20]. Through social media platforms, youth are connected to and are able to witness the beliefs and behaviors of a larger group of peers [21], where normative drug use may be featured and cultivated on the Web [22]. From a developmental perspective, adolescence is characterized by heightened attention to social norms and an increased desire for social approval [23,24]. Online discussions about drugs may be particularly impactful for this population as public posts can convey normative beliefs to other youth, particularly when posts support or promote drug use. Thus, the broadcasting of beliefs and behaviors related to drug use may influence youths’ perceptions of normative behavior, thereby influencing decisions on drug use among youth who are exposed to such conversations.

Although previous research has examined social media usage by youth as it relates to health-related outcomes [17,25], few studies have explored how youth discuss content related to substance use on Twitter. In one study, researchers examined the relationship between young adults’ alcohol-related tweets and self-reported cognitions and behaviors related to alcohol use; findings demonstrated that the proportion of one’s overall tweets related to alcohol was significantly associated with the willingness to drink and use alcohol [26]. In a separate study, researchers surveyed young adults about their exposure to alcohol- and marijuana-related content on Twitter and their use of these substances [21]. Analyses demonstrated significant associations between current heavy episodic drinking and higher levels of exposure to proalcohol content and between current marijuana use and higher levels of exposure to promarijuana content. However, no previous research has specifically examined the content of youths’ tweets about substance use, a potential predictor of beliefs and behaviors about substance use.

### Objectives

The primary goal of this study was to identify youths’ beliefs and behaviors related to drug use by characterizing the content of drug-related tweets by youth. Using a mixed methods approach, we investigated the relationships between the type of drug, language of the tweet, and reasons for drug use. This study provides insight into publicly stated beliefs about drugs and drug use on social media. Given the increased salience of social considerations (eg, social norms and external validation) during adolescence, youths’ tweets about substance use may contribute to the perception of what is *normal*, leading youth to espouse distorted perceptions of normative behavior and to model that behavior in real life. Through this systematic examination of youths’ beliefs about substance use, we can better understand the potential mechanisms driving substance use behavior.

## Methods

### Overview

This study employed a *sequential explanatory mixed methods* design to examine how popular drugs are discussed by youth on Twitter [27]. A mixed methods research approach was most appropriate given that quantitative or qualitative data, by themselves, would be insufficient to capture the nuances of youths’ tweets about substances. The use of mixed methods in social media research has grown in popularity as researchers seek to capitalize on the strengths of each data source [28], gaining a better understanding of their phenomena of interest. We chose the sequential explanatory design, placing priority on the qualitative data, to obtain a general understanding of the tweets and to contextualize the results.

**Figure 1:**
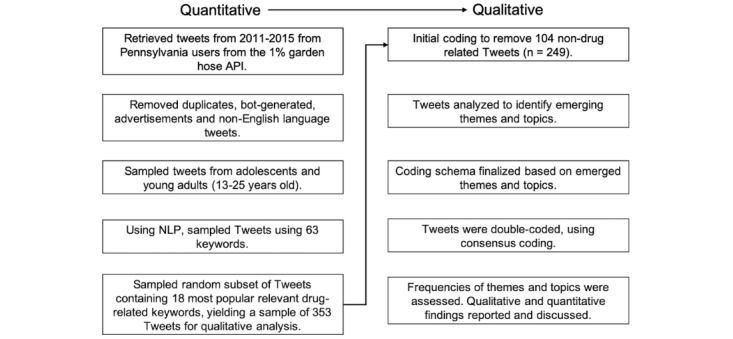
Data flow diagram. API: application programming interface; NLP: natural language processing.

Following this approach, we first conducted quantitative data collection and analysis to identify an appropriate sample of youths’ tweets, and then, we conducted qualitative data analysis to examine the content of these tweets. We used natural language processing (NLP) techniques to determine the frequency of use of drug-related words in public English-language tweets among the youth and emerging adult users of Twitter in Pennsylvania. We subsequently conducted a qualitative content analysis of a random sample of tweets for in-depth exploration of the context in which the words were used. This methodology provides a more nuanced view of substance use–related messages posted publicly by youth online, offering insights that may not be apparent through the analysis of quantitative or qualitative data in isolation. A key step in the mixed methods research is the integration of quantitative and qualitative data. We integrated data in two ways: (1) *connecting*, wherein the data were linked through the sampling frame such that we had quantitative and qualitative data for each participant, and (2) *merging*, wherein the two datasets were brought together for analyses [29]. For interpretation and reporting, we used a weaving approach whereby the NLP and content analysis results are presented together on a theme‐by‐theme basis [29]. See Figure 1 for the data flow diagram. This study was deemed exempt by the institutional review board of the University of Pennsylvania.

### Twitter Dataset

Data analysis was conducted with a sample of drug-related tweets posted on Twitter over a 4-year period. Using Twitter’s application programming interface, which provides broad access to public Twitter data, we drew a random sample of 1% of publicly available tweets posted between 2011 and 2015. Tweets were geolocated to US counties using tweet-specific latitude and longitude coordinates and self-reported location information in Twitter’s user profiles [30]. Using the open source Python package TwitterMySQL [31], we pulled the most recent 3200 tweets for each user geolocated to Pennsylvania, resulting in a dataset of over 440 million tweets. After removing both non-English tweets and duplicate tweets [32,33], often from bots and advertisers, we produced age and racial affiliation estimates for each user based on our tested algorithms [34,35]. We limited our sample by predicted age (13-17 years) and predicted race (black or non-Hispanic white), yielding 10,056 distinct adolescent users. We then randomly sampled a comparable number of emerging adult users with a predicted age of 18 to 24 years to match the adolescent sample. This approach yielded approximately 23 million tweets from 20,112 adolescents and young adults in Pennsylvania.

### Quantitative Retrieval of Drug-Related Tweets

To identify drug-relevant Twitter posts, we built lists of drug-related words and phrases, drawing from previous research [21], music lyrics, and slang dictionaries. We used these words and phrases to develop our classifier, which we iteratively improved using manual coders to assess the yields on a training dataset. Once we finalized the keyword list, comprising 63 drug-related words, we retrieved a random sample of tweets containing those keywords, yielding approximately 872 tweets for manual coding. The research team then identified which of the 63 keywords led to the retrieval of the largest number of relevant tweets. The 12 keywords that yielded the greatest proportion of drug-relevant tweets are listed in Table 1. We separated hashtagged versions of these words as well as word extensions (eg, “high” vs “highlife”), expanding our list to 18 keywords to capture potentially nuanced differences in usage.

We then selected a random sample of approximately 20 tweets containing each of the 18 frequently used drug-related keywords, totaling 353 tweets, for qualitative coding. As some keywords did not yield 20 relevant tweets, we coded the total number of tweets that were retrieved. Each tweet was coded independently by 3 coders to ensure it was drug-related (n=249). When coders could not reach agreement based on independent coding, they worked together to make a final determination. Nonrelevant tweets (n=104) were excluded from further analyses.

**Table 1:** Drug-related keywords, number of tweets sampled by keywords, relevant tweets, and keyword sensitivity.

Drug-related keywords	Tweets sampled by keyword (n=353), n	Relevant tweets (n=249), n	Keyword sensitivity^a^, n (%)
#blunt(s)	19	13	13 (68)
Blunt	20	12	12 (60)
#high	20	13	13 (65)
#highlife	20	12	12 (60)
High	20	6	6 (30)
#marijuana	14	7	7 (50)
Marijuana	20	12	12 (60)
#wakenbake	16	9	9 (56)
Ganja	20	17	17 (85)
Pot	20	10	10 (50)
Pothead	20	17	17 (85)
Smoke	20	18	18 (90)
Stoned	20	17	17 (85)
Stoner	20	13	13 (65)
#stoner	12	9	9 (75)
Weed	20	19	19 (95)
Adderall	20	16	16 (80)
Valium	12	11	11 (91)
Xanax	20	18	18 (90)

^a^Keyword sensitivity is the percentage of tweets from the total sample that were deemed relevant during manual coding.

### Qualitative Coding of Tweets Using Content Analysis

In the second stage of our mixed methods approach, we analyzed the sample of 249 drug-related tweets using established procedures for qualitative content analysis [36,37], a technique for making replicable and valid inferences from texts to the contexts of their use [22]. A total of 3 undergraduate students were extensively trained to code tweets for emergent themes. The relevant tweets were qualitatively analyzed in a multistage process that began with the identification of initial codes generated from prior literature and emerging themes. The team then coded the tweets until they reached consensus on thematic coding. Themes are not mutually exclusive; thus, tweets could be categorized under multiple themes. Inter-rater reliability was achieved at kappa=0.80 across key themes and topics, demonstrating acceptable reliability. We also calculated frequencies to describe the proportion of the sample categorized under each theme. In addition to emerging themes, we identified frequently occurring topics in tweets related to drug use and determined the proportion of tweets with antidrug content. All example tweets cited in the Results section were modified to retain their meaning, although they were rendered unsearchable on the internet [38]. The example tweets are accompanied by explanatory text, where appropriate, in brackets.

## Results

### Quantitative Findings

Table 1 lists the drug-related keywords used to retrieve the random sample of tweets and the number of tweets from each keyword included in the analysis. In total, we coded 249 tweets. As most of our keywords were specific to marijuana (12 out of 18), the majority of tweets in our analysis were also marijuana-related. Of the 6 keywords not explicitly related to marijuana, 3 were related to the prescription drugs Xanax, Percocet, and Adderall, and 3 keywords were nondrug-specific words related to substance use (“high,” “#high,” and “highlife”). We assessed the sensitivity of each keyword in retrieving relevant tweets. “Weed” was the most sensitive keyword, with the highest rate of retrieving drug-related tweets. “High” was the least sensitive keyword, retrieving relevant tweets 30.0% of the time.

We identified two broad classes of themes in our sample of tweets: *functional* themes and *relational* themes. Functional themes included posts that explicated a function of drugs in the user’s life. Within this broader classification, we identified functional subthemes indicative of *pride*, *longing*, *coping*, and *reminiscing* as they relate to drug use or effects. The second class of themes emphasized a relational nature of substance use. Specifically, this theme captured substance use as a part of social relationships. The subthemes identified in this category were *identity* and *companionship* as they relate to drug use. In addition to emergent themes, we also identified several topical areas in tweets related to drug use, which included reference to polysubstance use, pop culture, and antidrug content.

Across the sampled tweets, subthemes of *pride* (63/249, 25.3%) and *longing* (39/249, 15.7%) were the most popular. Thematic differences emerged between tweets about prescription drugs versus nonprescription drugs.

### Functional Themes

#### Pride

As the most popular theme, approximately one-quarter of all tweets in the sample (63/249, 25.3%) expressed pride in drug use. Tweets such as “This was the #first #blunt I rolled that the mans didn’t have to fix :) #wassoproud #smokeditsonice” and “Happy 420 !(: I just rolled my first blunt !! #RolledBlunt #Happy420” exemplify users’ pride in their skills related to drug use. Most tweets that expressed *pride* (46/63, 73%) were explicitly related to marijuana.

#### Longing

In tweets related to longing, users expressed yearning, desire, or craving for either a drug or the effects of a drug. Tweets such as “I would rather be high right now” and “could really use me a #blunt #like #now” explicitly portray the user’s desire for a substance or for the associated feeling. Although these tweets do not specify the actual intention or use of drugs in the future, the users communicated a desire to get high given their current circumstance.

#### Coping

Tweets related to coping described drug use as a coping strategy, often as a means to manage stressors and emotions, for example, “marijuana is useful for treating _____ INSERT whatev er the fuck your problem is here” and “Who ever said can’t buy happiness obviously didn’t kno w any pot dealers.” In addition, many coping-related tweets were about prescription drugs: “If it was not for adderall idk [I don’t know] how would deal with all of this college work rs [real shit].” Coping-related tweets are distinguished from longing-related tweets (eg, “I could use some Adderall right now...”) to the extent that the users specifically stated that they were trying to manage a condition (eg, a stressor or emotion) with drugs. Nearly half of the tweets on *coping* (17/36, 47%) were related to prescription drugs. Very few of the tweets contained *antidrug* content (9/249, 3.6%).

#### Reminiscing

Reminiscing described tweets that expressed nostalgia or wistfulness or depicted a user looking back at a drug-related experience. The tweet “That night I was soo drunk...soo high...Can’t even remember...#trippinBalls #MissThoseNights #HighLife” exemplifies how the user was fondly thinking back to a time when they used drugs. In the tweet “@[another user] yo go to the gram and look at that ganja i had last night lil,” the user is remembering and referring to their past drug use posted on Instagram, another social media platform.

### Relational Themes

#### Identity

Tweets were categorized under the identity category if the user classified himself/herself or another person with a drug-related label or name. For example, “She called me a pothead tho...Naaaah, I prefer stoner” labels the user as a pothead or a stoner. In the tweet “You know your boyfriends a pothead when he wakes up out of a dead sleep to smoke,” the user has categorized another drug user as a pothead. In this class of tweets, these names do not necessarily carry a negative connotation; rather, they convey the user’s pride in being thought of as a *stoner*.

#### Companionship

Tweets were categorized under the companionship theme if they expressed a feeling of fellowship or friendship, particularly when the tweet suggested that the user was looking for a companion to join in drug use. “Someone find me a #blunt and a cuddlebuddy” illustrates a Twitter user who indirectly asked the public for a companion to smoke with. “Burn riiiide with new friends???????? #bong #blunt #lovelife” portrays a different form of companionship where the user is not directly searching for a companion but describes the feeling of using drugs in fellowship with friends. The tweets in this category refer to the social connectedness component of substance use.

### Associated Topics

#### Polysubstance Use

Tweets within this category contained content that implied the use of multiple substances. “Got that percaset, promenthasen with codeine, Xanax!” suggests that the user intends to take or sell the 3 drugs mentioned with codeine. Another tweet, “I like to chase a few xanax bars with A crown royal” more clearly models polysubstance use, with the user explaining the order in which they prefer to use alcohol and another substance.

#### Pop Culture

Tweets containing content that referred to song lyrics, celebrities, or trending topics were categorized under pop culture. For example, “Ain’t a fucking sing along unless you brought the weed along” and “One by one, load up de van, all of a ganja it ram” are music lyrics. Although the tweets may or may not refer to the user’s actual drug-related behavior, these examples demonstrate how references to drug use in song lyrics are disseminated on social media.

#### Antidrug

Of the 249 tweets in our sample, only 9 (3.6%) included antidrug messaging. Antidrug tweets contained only 4 drug-related keywords: “marijuana,” “smoke,” “weed,” and “valium.” One such tweet conveys a strong antidrug perspective: “I see so many people of our generation glorifying xanax and valium and perks. It’s so fucking disgusting.” In another tweet, “No amount of weed is worth a fucking life,” the user links marijuana use with unspecified, though serious, repercussions.

### Themes and Drug-Related Keywords

**Figure 2:**
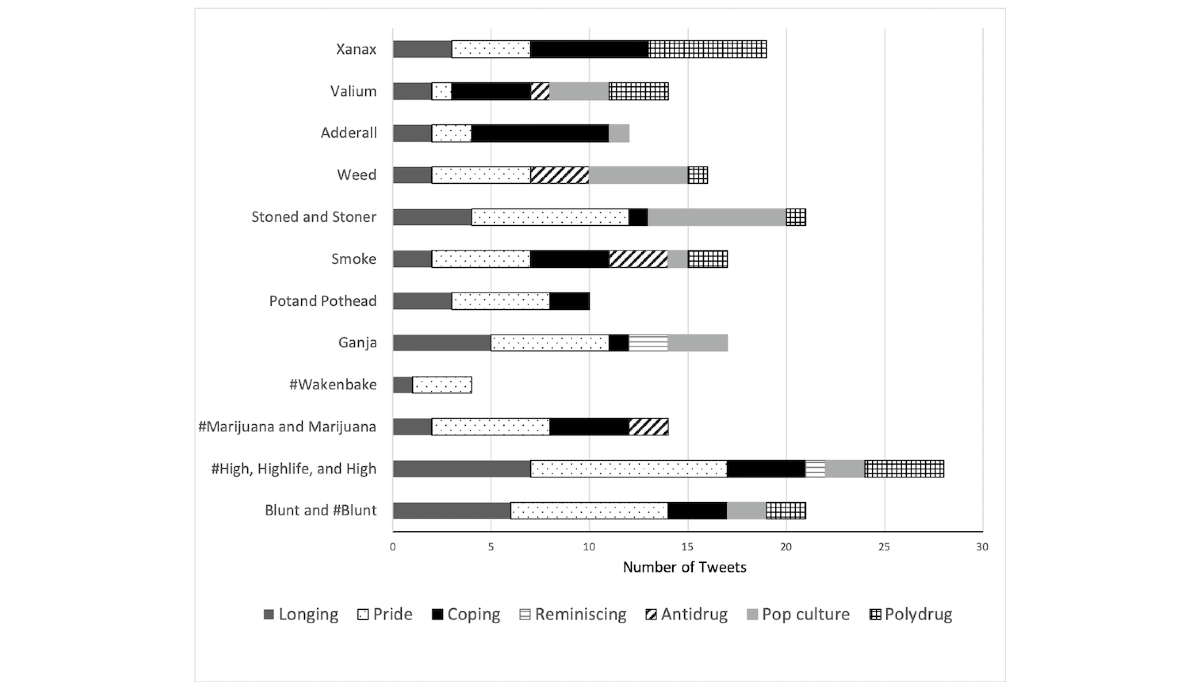
Frequency of themes from tweets having drug-related keywords.

Figure 2 is a visual representation of the presence of themes for each drug-related keyword. Except for Adderall, Valium, and Xanax, all drug-related keywords were most frequently associated with *pride, identity,* and *companionship*. Tweets about prescription drugs (Adderall, Valium, and Xanax), however, were more frequently categorized under the themes of *coping* and *polysubstance use*.

## Discussion

### Principal Findings

Youths’ discourse about drug use on Twitter offers valuable insights into the normative beliefs and behaviors, as expressed online. Our systematic mixed methods approach to examine the use of Twitter by youth to discuss drugs furthers our understanding of the potential mechanisms driving substance use behavior, from an expression of pride to a means for coping with life stressors. In the tweets studied, we found that the most popular drug-related keywords were related to marijuana, followed in popularity by prescription drugs. The relative popularity of drug-related keywords related to these substances mirrors broader substance use patterns as marijuana is the illicit substance that is most commonly used among youth [1]. Thematic analyses indicate that drugs were typically discussed in a positive manner, including positive messages about previous experiences with drugs or one’s desire to use drugs again. Our findings also suggest that users are comfortable posting public endorsements of drug use.

Youth expressed pride, confidence, or boastfulness online about their drug-related behaviors. Youth who boasted about their drug use on Twitter often linked drug use to their identities. In addition, online discussions of drug use were regularly associated with social contexts, mirroring the correlation of youths’ substance use in offline settings. In many tweets, youth indicated a craving or desire for a drug or the effects of drug use. This is particularly notable as there was little discussion about the addictive nature of substances. Without this, the risks and negative outcomes associated with drug use are largely absent from peer online discussion.

We found that prescription drugs were used for coping, specifically as a tool to cope with challenges, grief, or stress. These tweets may help explain, in part, the rise in misuse of prescription drugs, with approximately 2200 youth misusing pain medications each day [2]. Youth online view Xanax, Percocet, and Valium as tools to cope with the challenges they face rather than as a part of peer social drug use (as was seen with marijuana). When youth opt to use these drugs for emotional regulation and to help deal with life stressors, they can increase their risk of future addiction [39]. The tweets also reveal that prescription drug–related tweets are mentioned along with other drugs and alcohol use. This echoes previous research, which found that almost 1 in 10 Adderall-related tweets contained reference to another substance [40].

Substance use among youth is a highly social behavior to the extent that the usage patterns are influenced heavily by perceived peer norms and behaviors [23]. Substance use messages posted on social media are related to youths’ substance use behaviors offline and may also influence the normative beliefs of youth who are exposed to those messages [41-44]. When youth describe the frequency of their marijuana or prescription drug use online, these messages endorse substance use as normative behavior among youth. This holds true despite the potential legal implications of underage drinking or illegal substance use. For example, in Pennsylvania, recreational marijuana use is illegal and medical marijuana use is highly regulated. However, on social media, youth discuss and disclose their drug use behavior, although these behaviors are illegal in the state. Thus, the perceived norm of substance use acceptability may outweigh the perceived consequences of such use.

Although our analysis uncovered posts about the negative consequences or effects of substance use, as demonstrated in previous research [16,45-48], these posts represented less than 3.6% (9/249) of our sample. In the absence of such antidrug messages, social media platforms may convey a meta-message to youth that the usage of drugs, specifically marijuana, is not associated with adverse consequences. It is notable that prescription drugs were not discussed with the same level of pride as marijuana. However, prescription drug–related posts often included reference to other substances, suggesting that the discussions of prescription drug use on social media are an indicator of polysubstance use. Strategies that address the underlying causes of drug use (eg, coping with stressors) and engage the positive drug messaging on social media are needed to help reduce the elevated prevalence of early polysubstance use behavior among adolescents [22].

### Limitations

There are several limitations to this study. The subset of tweets we examined may not represent the entire population of youths’ tweets containing drug-related content; thus, our results may not generalize beyond the study sample. It is possible that additional drug-related keywords were missed in the culling of the data and are thus missing from analyses. Social desirability may bias results, leading youth to post specific prodrug content such that they appear to endorse substance use beliefs and behaviors online that they may not actually hold. Moreover, the cross-sectional study design limits our ability to link the tweets to actual offline substance use behavior. Future longitudinal studies are needed to examine youths’ social media posts overtime, correlating these posts with substance use–related behaviors and identifying predictors of future drug use based on social media use behavior.

### Conclusions

With its great popularity among youth, social media is a fruitful platform for examining youth cognitions and behavior related to specific drug use. Through a mixed methods approach, we established the frequency with which drugs are discussed by members of this population on Twitter, generated a list of words and hashtags to contribute to analytical lexicons for others interested in similar research, and identified themes indicative of the ways in which youth discuss their support for (or opposition to) substance use on social media. Together, these findings contribute to the literature by indicating a critical need to leverage social media to challenge myths and unhealthy online substance use norms. Further inquiry is needed to better understand how exposure to drug-related content on social media influences youths’ behavior and to identify ways to leverage positive aspects of social media (eg, group connectedness and sharing of health-related information) to decrease substance use and improve health outcomes.

## Abbreviations

NLP: natural language processing
